# Explorative Binary Gray Wolf Optimizer with Quadratic Interpolation for Feature Selection

**DOI:** 10.3390/biomimetics9100648

**Published:** 2024-10-21

**Authors:** Yijie Zhang, Yuhang Cai

**Affiliations:** School of Artificial Intelligence and Computer Science, Jiangnan University, Wuxi 214122, China; 8202101464@jiangnan.edu.cn

**Keywords:** gray wolf optimizer, feature selection, quadratic interpolation, classifier, evolutionary computation

## Abstract

The high dimensionality of large datasets can severely impact the data mining process. Therefore, feature selection becomes an essential preprocessing stage, aimed at reducing the dimensionality of the dataset by selecting the most informative features while improving classification accuracy. This paper proposes a novel binary Gray Wolf Optimization algorithm to address the feature selection problem in classification tasks. Firstly, the historical optimal position of the search agent helps explore more promising areas. Therefore, by linearly combining the best positions of the search agents, the algorithm’s exploration capability is increased, thus enhancing its global development ability. Secondly, the novel quadratic interpolation technique, which integrates population diversity with local exploitation, helps improve both the diversity of the population and the convergence accuracy. Thirdly, chaotic perturbations (small random fluctuations) applied to the convergence factor during the exploration phase further help avoid premature convergence and promote exploration of the search space. Finally, a novel transfer function processes feature information differently at various stages, enabling the algorithm to search and optimize effectively in the binary space, thereby selecting the optimal feature subset. The proposed method employs a k-nearest neighbor classifier and evaluates performance through 10-fold cross-validation across 32 datasets. Experimental results, compared with other advanced algorithms, demonstrate the effectiveness of the proposed algorithm.

## 1. Introduction

With the rapid advancement of information technology, the volume of data generated daily is increasing exponentially, and people have begun to realize the vast amounts of valuable information and knowledge contained within these data. Data mining has thus emerged as a crucial tool for extracting valuable information from massive datasets. In recent years, data mining has become an indispensable field, widely applied in finance, healthcare, bioinformatics, and other areas. Through data mining techniques, valuable information and knowledge hidden within large datasets can be uncovered, providing decision support and competitive advantages for enterprises. However, despite its high rewards, data mining faces numerous challenges and difficulties. As the dimensionality of data increases, the size of the data space grows exponentially, leading to significant increases in computational and storage costs. High-dimensional data not only increase the complexity of algorithms but also extend model training time and can lead to the curse of dimensionality, characterized by decreased sample density and difficulty in distance calculations. Furthermore, the quality of collected datasets is often uneven and may be accompanied by high levels of noise, missing values, and other issues that affect the accuracy and reliability of models. To effectively utilize data mining, data preprocessing is applied to clean and prepare data for the next stage of machine learning, aiming to eliminate unnecessary and redundant features from large datasets [1]. This process reduces data dimensionality, thereby improving the generalization capability and accuracy of models [2]. In machine learning classification problems, when a feature subset contains redundant features, removing these features does not increase the classifier’s error rate; however, when all features in the subset are crucial, removing any one of them may reduce the classifier’s accuracy. Therefore, feature selection, as a necessary preprocessing technique in machine learning classification problems, primarily aims to select the optimal subset of features that best represent the data’s attributes while improving classification accuracy on the dataset. Consequently, feature selection is typically modeled as a multi-objective optimization problem, encompassing two objectives: the number of selected features and classification accuracy.

There are many feature selection methods that can help choose the optimal feature subset, generally categorized into three types: filter methods, wrapper methods, and embedded methods. Filter methods evaluate each feature independently of any learning algorithm, ranking or selecting features based on a specific evaluation metric. In contrast, wrapper methods evaluate feature subsets by using a specific learning algorithm to train a model and select feature subsets based on the model’s performance. This approach decides which features should be retained or removed and is more suited to practical application scenarios, especially classification problems. Wrapper methods are usually more accurate than filter methods but are also more computationally intensive, as they require repeatedly training models to evaluate the performance of each feature subset.

However, most current feature selection methods still have limitations when dealing with large-scale high-dimensional data. Although wrapper methods generally offer high accuracy, they come with significant computational costs. Even with recursive and sequential feature selection methods, it remains challenging to cope with the rapid growth in dataset dimensions. Therefore, reducing computational complexity while maintaining classification accuracy remains a pressing research problem.

In recent years, metaheuristic algorithms have made substantial progress in various fields, particularly in optimization. The ability of metaheuristic algorithms to find the globally optimal solution within a reasonable time makes them well suited for searching for the best feature subsets [3]. However, most traditional metaheuristic algorithms suffer from insufficient population diversity, a tendency to get trapped in local optima, and weak exploration capabilities.

This paper proposes the EQIGWO algorithm, which significantly improves upon the existing Gray Wolf Optimization (GWO) algorithm. Compared to traditional optimization algorithms, the EQIGWO algorithm exhibits excellent global search capabilities and convergence precision and reduces the risk of becoming stuck in local optima, making it particularly suitable for feature selection tasks. The novelty of this study lies in several key aspects. First, the EQIGWO algorithm enhances exploration capabilities by integrating individual information. Second, it improves population diversity and local exploitation by incorporating quadratic interpolation techniques for local development. Third, chaotic perturbation is introduced during the exploration phase to further enhance global exploration capabilities. Finally, a novel transfer function is employed, which makes the algorithm particularly effective for the binary nature of feature selection tasks. Additionally, the optimization performance of the EQIGWO algorithm has been validated through a series of experiments, demonstrating its superior performance across multiple datasets.

The organization of this paper is as follows. Section 2 discusses related works on feature selection. Section 3 provides a description of the standard Gray Wolf Optimizer algorithm. In Section 4, the proposed algorithm (EQIGWO) is elaborated on in more detail. Section 5 presents the numerical results and comparisons. Finally, Section 6 offers some conclusions.

## 2. Related Works

In recent years, metaheuristic algorithms have been widely applied and researched in the context of the multi-objective optimization problem of feature selection. For example, the Bacteria Foraging Optimization Algorithm (BFOA), proposed by Das et al., seeks the optimal solution by moving toward regions with higher nutrient concentrations. It has been widely applied to feature selection problems to enhance global search capability and accuracy [4]. The Fruit Fly Optimization Algorithm (FOA), introduced by Xing et al., offers a feature selection method with lower computational complexity and faster convergence speed, making it particularly suitable for feature selection in high-dimensional datasets [5]. Additionally, the Virus Evolutionary Genetic Algorithm (VEGA), proposed by Ling et al., combines virus evolution mechanisms with genetic algorithms, enhancing the diversity and adaptability of the search process. In feature selection tasks, the VEGA demonstrates strong global search ability and improved optimization performance [6]. Zhang et al. proposed a novel Harris Hawk Optimization (IHHO) algorithm, which embeds the Salp Swarm Algorithm (SSA) into the original HHO algorithm. The SSA is used to adjust the population to generate an SSA-based population, create hybrid individuals based on both SSA and HHO individuals, and update search agents according to greedy selection and HHO mechanisms. However, the algorithm’s performance is highly dependent on parameter settings, requiring experimentation to determine the optimal configuration [7]. Tu et al. proposed a multi-strategy integrated Gray Wolf Optimizer for feature selection (MEGWO). MEGWO employs three different search strategies to update solutions. First, an improved global optimal guidance strategy exploits the search space around the current optimal solution, enhancing local search capabilities. Second, an adaptive cooperative strategy embeds one-dimensional update operations into the GWO framework, providing higher population diversity and improving global search capabilities. Third, a dispersed foraging strategy forces some search agents to explore promising regions based on a self-adjusting parameter, balancing exploration and exploitation [8]. Hu et al. analyzed the value range of the AD parameter under binary conditions and proposed a new α parameter update equation to balance global and local search capabilities. They also introduced five transfer functions to convert continuous values to binary values, successfully achieving feature selection in UCI datasets with a low classification error and a small number of features [9]. Hu et al. also proposed a fuzzy cost-based multi-objective particle swarm optimization method for feature selection (PSOMOFS). They developed a fuzzy dominance relation to compare the quality of candidate particles and defined a fuzzy crowding distance measure to trim the elite archive and determine the global leader of the particle swarm. Additionally, a tolerance coefficient was introduced to ensure a solution set that allows for selection based on practical application needs, increasing the applicability and flexibility of the solutions [10]. Furthermore, Hu et al. improved the original Gray Wolf Optimizer by developing an enhanced variant (GWOCMALOL) based on the Covariance Matrix Adaptation Evolution Strategy (CMA-ES), Levy flight mechanism, and orthogonal learning strategy. The algorithm generates a set of orthogonal samples through orthogonal learning to preserve and effectively search for optimal direction information, aiding in faster convergence to the global optimum. The Levy flight strategy enhances exploration by widely distributing individuals in the target space, improving global search capabilities. CMA-ES addresses the issue of the algorithm focusing solely on exploitation in the later stages [11]. Zhang et al. proposed a binary differential evolution algorithm with an adaptive learning strategy named MOFS-BDE for multi-objective feature selection. They introduced a novel probability difference-based binary mutation in the algorithm to improve convergence without sacrificing global search capability. By repeatedly applying a one-bit purifying search on elite individuals to generate multiple new individuals, the quality of non-dominated solutions in the population was enhanced. They also embedded an efficient non-dominated sorting operator with crowding distance in the algorithm to reduce the time consumption of the selection operator in differential evolution [12]. Jin et al. proposed a novel approach that combines traditional Boids modeling, inspired by bird swarm behavior, with advanced deep reinforcement learning techniques to enhance the performance of UAVs in pursuit-evasion scenarios. By combining Boids with DRL, UAVs are empowered with both adaptive, real-time decision-making capabilities and the natural, efficient movement patterns seen in bird swarms. This hybrid model allows UAVs to dynamically adjust their behavior based on real-time environmental feedback, leading to improved cooperative and evasive strategies [13]. Additionally, Xia et al. introduced an enhanced version of the Moth-Flame Optimizer by incorporating quasi-reflection and refraction learning mechanisms to augment the algorithm’s global and local search capabilities. Quasi-reflection learning increases the diversity of the population and expands the search space during iteration jumps. Innovatively, the concept of refraction is introduced to adjust the algorithm’s search behavior, thereby improving the quality of solutions and search efficiency [14]. Pan innovatively uses a two-step optimization method to strategically allocate new healthcare resources. The first step utilizes a spatial optimization model to optimize hospital locations, aiming to maximize healthcare service coverage for the population. In the second step, capacity is allocated to each hospital to ensure equitable access to healthcare services across different residential locations. This approach effectively balances the goals of maximizing coverage and ensuring fairness in healthcare access [15].

## 3. Gray Wolf Optimization

In the Gray Wolf Optimizer (GWO), the three wolves with the best fitness are defined as the three leaders: alpha, beta, and delta wolves. The other wolves are designated as omega wolves. The omega wolves surround the prey and hunt under the guidance of the three leaders. Each gray wolf represents a candidate solution, and the position vector of a gray wolf corresponds to the feature vector of the candidate solution. The objective function value of the candidate solution corresponds to the fitness of the gray wolf.

To model the encircling behavior of gray wolves while hunting prey, the following equations have been proposed:

(1) $$\vec{D}=\left|\vec{C}\;\cdot \;\vec{X_{p}}\left(t\right)-\vec{X}\left(t\right)\right|$$

(2) $$\vec{X}\left(t+1\right)=\vec{X_{p}}\left(t\right)-\vec{A}\;\cdot \;\vec{D}$$

(3) $$\vec{\alpha }\left(t\right)=2-\frac{2t}{MaxIter}$$

(4) $$\vec{A}=2\vec{\alpha }\;\cdot \;\vec{r_{1}}-\vec{\alpha }$$

(5) $$\vec{C}=2\;\cdot \;\vec{r_{2}}$$

where *t* represents the current iteration number, MaxIter is the total iteration number, $\vec{D}$ is the distance between the wolf and the prey, $\vec{X_{p}}\left(t\right)$ is the position vector of the prey, $\vec{X}\left(t\right)$ is the position vector of the wolf at iteration t, $\vec{A}$ and $\vec{C}$ are coefficient vectors, $\vec{r_{1}}$ and $\vec{r_{2}}$ are random vectors in [0, 1], and the component of *a* decreases linearly from 2 to 0 during the iteration process.

The omega wolves hunt under the guidance of the three leader wolves. The equation for this behavior is as follows:

(6) $$\vec{D_{\alpha }}=\left|\vec{C_{1}}\;\cdot \;\vec{X_{\alpha }}-\vec{X}\right|,\;\vec{D_{\beta }}=\left|\vec{C_{2}}\;\cdot \;\vec{X_{\beta }}-\vec{X}\right|,\;\vec{D_{\delta }}=\left|\vec{C_{3}}\;\cdot \;\vec{X_{\delta }}-\vec{X}\right|$$

(7) $$\vec{X_{1}}=\vec{X_{\alpha }}-\vec{A_{1}}\;\cdot \;\vec{D_{\alpha }},\;\vec{X_{2}}=\vec{X_{\beta }}-\vec{A_{2}}\;\cdot \;\vec{D_{\beta }},\;\vec{X_{\delta }}=\vec{X_{\delta }}-\vec{A_{3}}\;\cdot \;\vec{D_{\delta }}$$

(8) $$\vec{X}\left(t+1\right)=\frac{\vec{X_{1}}+\vec{X_{2}}+\vec{X_{3}}}{3}$$

where $\vec{X_{\alpha }}$, $\vec{X_{\beta }}$, and $\vec{X_{\delta }}$ are the position vectors of the alpha, beta, and delta wolves.

## 4. The Proposed Algorithm

As described in Section 3, the standard GWO tends to fall into local optima during the feature selection process, especially in high-dimensional feature spaces where the algorithm may lack sufficient exploration ability, preventing it from finding the global optimum. In the later stages of iteration, population diversity may be insufficient, making it difficult for the algorithm to avoid local optimum traps and fully explore all potential solutions in the search space. In this section, the EQIGWO algorithm is proposed to tackle feature selection problems. A detailed explanation of each step is presented in the following subsections, and the complete pseudocode for the EQIGWO algorithm can be found in Algorithms 1 and 2.    
**Algorithm 1: **The EQIGWO
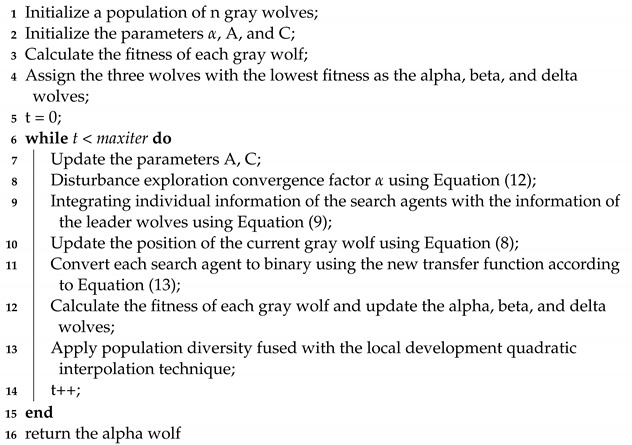


**Algorithm 2: **Population diversity fused with local development quadratic interpolation technique

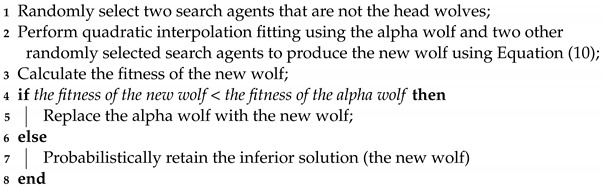



### 4.1. The Linear Integration of Individual Information Enhances the Exploration Strategy

**Figure 1:**
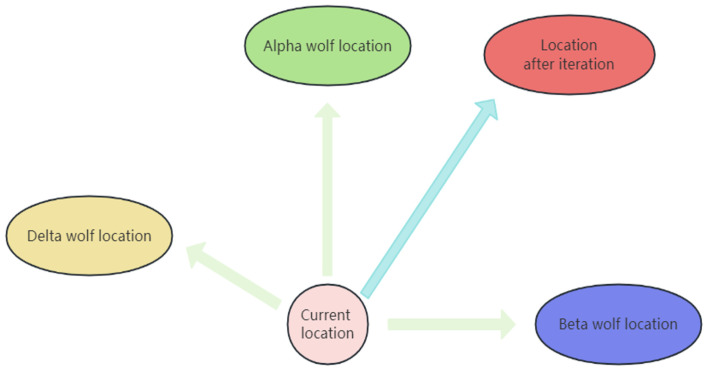
Position update in GWO.

**Figure 2:**
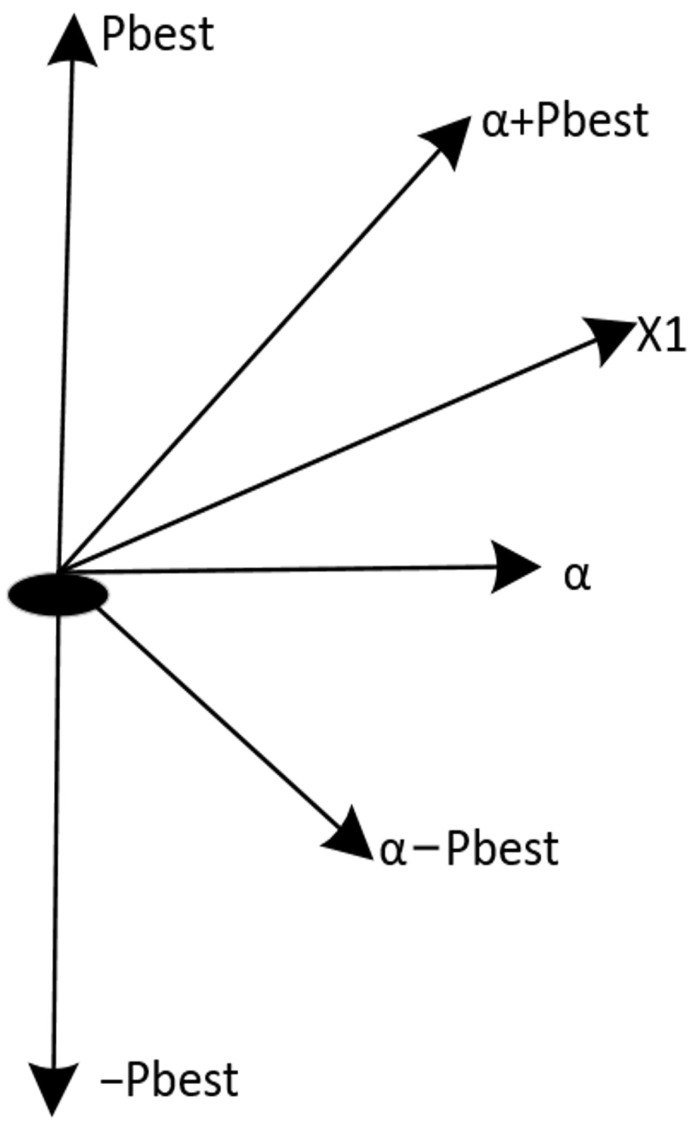
Position update in EQIGWO.

Through Equation (6) and Figure 1, we can observe that in the original GWO, the search direction of the search agents is entirely determined by the alpha, beta, and delta wolves due to their better understanding of potential prey positions. This results in the search agents slowly converging toward the three leader wolves. Consequently, in the later iterations of the algorithm, all search agents tend to cluster within a small range, engaging in inefficient exploitation and neglecting other regions of the search space that may potentially yield optimal solutions. This tendency makes the algorithm highly susceptible to falling into local optima. Furthermore, when facing complex optimization problems, the likelihood of the three leader wolves becoming trapped in local optima increases significantly. In such scenarios, due to the search agents’ reliance on the leader wolves, the algorithm tends to converge toward local optima prematurely, leading to decreased convergence accuracy. Additionally, the leadership nature of the GWO’s leader wolves also implies a lack of potential for escaping local optima. As depicted in Figure 2, by linearly integrating the individual information of the search agents with that of the leader wolves, the dependency on the leader wolves can be reduced. This integration facilitates more effective exploration of the search space, thus mitigating the occurrence of these issues. The specific equation for achieving this integration is as follows:

(9) $$\begin{array}{cc} \; & \vec{D_{\alpha }}=\left|\vec{C_{1}}\;\cdot \;\left(\frac{\vec{X_{\alpha }}+\vec{X_{pbest}}}{2}\right)-\vec{X}+\vec{C_{4}}\;\cdot \;\left(\frac{\vec{X_{\alpha }}-\vec{X_{pbest}}}{2}\right)-\vec{X}\right|\\ \; & \vec{D_{\beta }}=\left|\vec{C_{2}}\;\cdot \;\left(\frac{\vec{X_{\beta }}+\vec{X_{pbest}}}{2}\right)-\vec{X}+\vec{C_{5}}\;\cdot \;\left(\frac{\vec{X_{\beta }}-\vec{X_{pbest}}}{2}\right)-\vec{X}\right|\\ \; & \vec{D_{\delta }}=\left|\vec{C_{3}}\;\cdot \;\left(\frac{\vec{X_{\delta }}+\vec{X_{pbest}}}{2}\right)-\vec{X}+\vec{C_{6}}\;\cdot \;\left(\frac{\vec{X_{\delta }}-\vec{X_{pbest}}}{2}\right)-\vec{X}\right| \end{array}$$

where c1, c2, c3, c4, c5, and c6 are random vectors in the range [0, 1], and $\vec{X_{pbest}}$ represents the position vector of the individual’s historical best position.

Integrating the individual information of the search agents with that of the leader wolves helps increase population diversity and explore broader regions of the solution space. We utilize the historical best positions of the search agents to ensure the exploration of potentially fruitful regions. Moreover, relying too heavily on the leader wolves may lead to local convergence, even when integrating the historical best positions of the individuals. However, regions opposite to an individual’s best position may also contain optimal solutions. Therefore, we linearly combine the historical best positions and their opposite directions with the global best position to enhance information exchange within the population while ensuring exploration toward promising regions.

### 4.2. Population Diversity Fused with Local Development Quadratic Interpolation Technique

When dealing with complex datasets composed of high-dimensional features, the large scale of the search space and the reliance on the leader wolves make the algorithm prone to inefficient iterations in local regions, resulting in low convergence accuracy and susceptibility to local optima. Quadratic interpolation is a commonly used curve-fitting technique, often employed to find the minimum point of a univariate function within a given initial interval. Utilizing quadratic interpolation to fit the positions of the alpha wolf and two randomly selected search agents to generate candidate solutions for updating the position of the leader wolf can help improve the algorithm’s convergence accuracy. However, quadratic interpolation requires careful selection of three points; improper point selection may construct a parabola opening upwards, leading to maximization rather than minimization. The quadratic fitting in the algorithm is conducted dimension-wise; thus, it cannot guarantee that an opening-down parabola will be constructed in each dimension. This may result in candidate solutions that are inferior to the alpha wolf. However, inferior solutions can help the algorithm maintain diversity in the search space and avoid falling into local optima. By introducing a certain degree of randomness or accepting inferior solutions, the algorithm can better explore the entire search space rather than being limited to a local region. If the algorithm prematurely converges to a local optimum, it may miss better solutions. The existence of inferior solutions can aid the algorithm in continuing exploration until better solutions are found. Therefore, this paper probabilistically retains inferior individuals based on the difference in fitness values, as specified by the following equation:

(10) $$X_{new}^{j}=0.5\frac{\left[{\left(X_{r3}^{j}\right)}^{2}-{\left(X_{r4}^{j}\right)}^{2}\right]\;\cdot \;f\left(\vec{X_{\alpha }}\right)+\left[{\left(X_{r3}^{j}\right)}^{2}-{\left(X_{\alpha }^{j}\right)}^{2}\right]\;\cdot \;f\left(\vec{X_{r4}}\right)+\left[{\left(X_{r4}^{j}\right)}^{2}-{\left(X_{\alpha }^{j}\right)}^{2}\right]\;\cdot \;f\left(\vec{X_{r3}}\right)}{\left[\left(X_{r3}^{j}\right)-\left(X_{r4}^{j}\right)\right]\;\cdot \;f\left(\vec{X_{\alpha }}\right)+\left[\left(X_{r3}^{j}\right)-\left(X_{\alpha }^{j}\right)\right]\;\cdot \;f\left(\vec{X_{r4}}\right)+\left[\left(X_{r4}^{j}\right)-\left(X_{\alpha }^{j}\right)\right]\;\cdot \;f\left(\vec{X_{r3}}\right)}$$

(11) $$\left\{\begin{array}{c} if\;f\left(\vec{X_{new}}\right)<f\left(\vec{X_{\alpha }}\right)\;\vec{X_{\alpha }}=\vec{X_{new}}\\ elif\;\frac{e^{f\left(\vec{X_{\alpha }}\right)-f\left(\vec{X_{new}}\right)}}{2}>r_{6}\;\mathrm{insert}\;\vec{X_{new}} \end{array}\right.$$

We choose the alpha wolf and two other randomly selected search agents for quadratic interpolation fitting. The alpha wolf plays a guiding role in quadratic interpolation, enabling the candidate solutions to undergo a fine-grained search near the alpha wolf. By performing quadratic interpolation on the positions of the three search agents, a smoother surface can be fitted, and more promising search directions can be selected based on this surface. This helps improve the algorithm’s local convergence capability, enabling it to find solutions close to the global optimum. When a candidate solution is superior to the alpha wolf, we choose to directly replace the alpha wolf with the candidate solution to enhance the algorithm’s convergence accuracy. This method also helps the algorithm escape from local optima when it becomes trapped. When a candidate solution is inferior, we probabilistically retain it based on the difference in fitness values. Inferior solutions provide some path information about the search space, guiding the algorithm toward better directions. Although these solutions themselves may not be optimal, their presence helps the algorithm explore the search space more effectively. If the fitness of an inferior solution is poor, the probability of retention is low to avoid affecting the convergence speed of the algorithm.

### 4.3. Disturbance Exploration Convergence Factor

In GWO, the convergence factor α linearly decreases from 2 to 0 as the number of iterations increases. The process of decreasing the convergence factor α from 2 to 1 represents the gradual weakening of the algorithm’s exploration capability, which may lead to insufficient exploration in the early stages when facing complex problems, resulting in the algorithm easily becoming trapped in local optima and being unable to escape. To address this issue, this paper introduces chaotic perturbation to the convergence factor during the exploration phase, as shown below.

(12) $$\begin{array}{cc} \; & if\;t<\;T:\\ \; & \alpha \left(t\right)=2-2\times \left(t/\max iter\right)\times k\left(t\right)\\ \; & k\left(t\right)=4\times k\left(t-1\right)\times \left(1-k\left(t-1\right)\right) \end{array}$$

where *T* is the threshold set to 15.

By perturbing the convergence factor during the exploration phase, it exhibits good randomness rather than the original linear decrease, ensuring sufficient exploration in the early stages of the algorithm. The convergence factor after perturbation during the exploration phase is numerically higher than the original convergence factor, enhancing the algorithm’s global search capability. Especially in the mid to late stages of exploration, the original convergence factor diminishes the algorithm’s exploration capability almost entirely, while the perturbed convergence factor can still maintain a relatively large value, ensuring strong exploration ability and exploring regions unknown to the original algorithm, laying the groundwork for subsequent development stages.

### 4.4. New Transfer Function

The original Gray Wolf Optimization (GWO) algorithm is typically designed for continuous value problems rather than binary problems. However, in the context of feature selection, the problem revolves around selecting or not selecting (0 or 1) the most beneficial features to maximize classification accuracy. Therefore, when applying the GWO algorithm to feature selection, the transfer function plays a crucial role. It is primarily used to map continuous values in the feature space to the binary space, thereby achieving feature discretization. This transformation allows the algorithm to search and optimize in the binary space, effectively selecting the optimal feature subset.

The transfer function controls the probability of selecting or not selecting a particular feature, and an inappropriate transfer function may lead to a decrease in algorithm efficiency. This section proposes a novel piecewise probability selection transfer function, as shown below.

(13) $$\begin{array}{c} \begin{array}{cc} \; & when\;t\leq 15:\\ \; & \;if\;t/\max iter<rand:\\ \; & \;if\frac{1}{1+e^{-10(X_{j}-0.4)}}>rand\;\cdot \;4/5:\\ \; & \;X_{j}^{bin}=1\\ \; & \;else:\\ \; & \;X_{j}^{bin}=0\\ \; & \;else:\\ \; & \;if\frac{1}{1+e^{-10(X_{j}-0.5)}}>rand:\\ \; & \;X_{j}^{bin}=1\\ \; & \;else:\\ \; & \;X_{j}^{bin}=0 \end{array}\;\;\begin{array}{cc} \; & when\;t>15:\\ \; & \;if\;t/\max iter<rand:\\ \; & \;if\frac{1}{1+e^{-10(X_{j}-0.5)}}>rand:\\ \; & \;X_{j}^{bin}=1\\ \; & \;else:\\ \; & \;X_{j}^{bin}=0\\ \; & \;else:\\ \; & \;if\tanh\left(X_{j}\right)/\tanh\left(1\right)>rand:\\ \; & \;X_{j}^{bin}=1\\ \; & \;else:\\ \; & \;X_{j}^{bin}=0 \end{array} \end{array}$$

**Figure 3:**
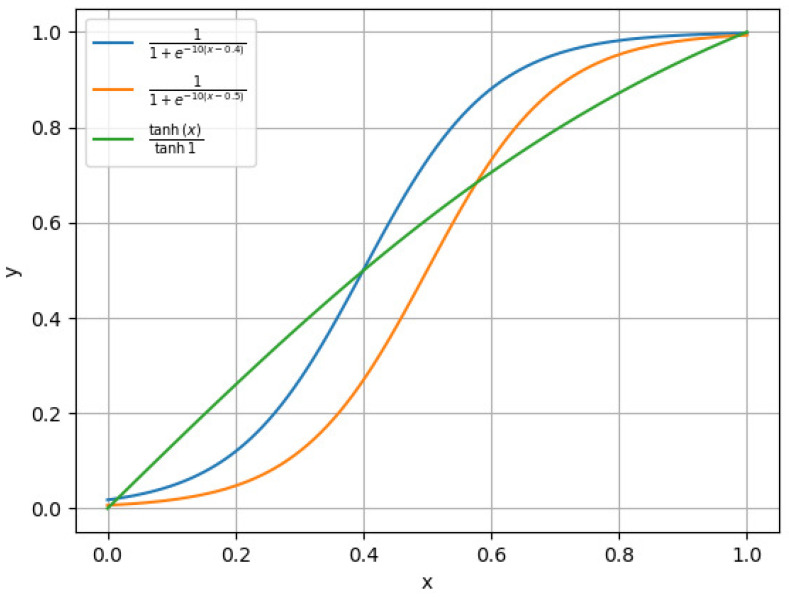
Plot of three functions.

In the early iterations of the algorithm, to prevent the loss of crucial feature information, $\frac{t}{\max iter}$ is relatively small, resulting in a higher probability of using the function $\frac{1}{1+e^{-10(X_{j}-0.4)}}$. As shown in Figure 3, $\frac{1}{1+e^{-10(X_{j}-0.4)}}$ remains consistently greater than $\frac{1}{1+e^{-10(X_{j}-0.5)}}$ over the interval [0, 1], ensuring that the values mapped from the continuous space to the binary space are relatively large, thereby maintaining a higher probability of retaining features.

In the mid-iterations of the algorithm, $\frac{t}{\max iter}$ gradually increases, and the function $\frac{1}{1+e^{-10(X_{j}-0.5)}}$ is used more frequently. This helps reduce the number of features and avoid meaningless additions, addressing the curse of dimensionality and achieving the purpose of feature selection.

In the later iterations of the algorithm, $\frac{t}{\max iter}$ has a larger value, allowing the algorithm to adjust the importance of current key features without affecting their retention through the function $\tanh\left(X_{j}\right)/\tanh\left(1\right)$, which has a larger value in the front segment and a smaller value in the back segment. This enhances the values of current non-key features in the binary space, increasing the probability of selecting these non-key features, thereby adding more informational content and improving classification accuracy.

## 5. Experimental Results and Discussion

All the coding in this paper was implemented on a Windows 10 platform using Python 3.8 on a computer with an Intel(R) Core(TM) i5-8300H CPU processor and 8GB of memory.

### 5.1. Evaluation Metrics

Feature selection is a multi-objective problem involving classification accuracy and the number of selected features. This article proposes a convenient evaluation function that combines classification accuracy and feature subset size in a weighted manner. If the evaluation function only considers classification accuracy, it may overlook solutions that have the same accuracy but fewer selected features. Therefore, integrating classification accuracy and the size of the feature subset can offer a more holistic feature selection evaluation metric, as shown below.

(14) $$f=a\times error+b\times \frac{s}{c}$$

where error represents the classification error from cross-validation, S denotes the length of the final selected feature subset, and *c* is the number of features per sample in the dataset. Parameters *a* and *b* are weights assigned to the importance of classification accuracy and the length of the selected feature subset, respectively. These parameters belong to the interval [0, 1], with *a* = 1 − *b*.

### 5.2. Description of Datasets

This section evaluates the performance of the proposed algorithm on 32 well-known datasets. The datasets are sourced from the UCI Machine Learning Repository [16], encompassing varying numbers of attributes and samples across diverse domains, and are also available online at https://www.openml.org (accessed on 20 May 2024), as illustrated in Table 1. For each dataset, information regarding the number of samples, features, and classes is provided.

### 5.3. Parameter Settings

The performance of the proposed algorithm was compared with 10 other advanced feature selection algorithms (BA [17], BCSA [18], bGWO [19], bWOA [20], DPSO [21], FA [22], MVO [23], PSO [24], NLPSO [25], and TMGWO [26]). The parameters of the comparison algorithms are shown in Table 2. All algorithms were run independently 20 times on each dataset using random seeds, with a maximum iteration of 30, five search agents, and parameters a and b in the evaluation function set to 0.99 and 0.01, respectively. The k-nearest neighbor (KNN) classifier is one of the most commonly used classifiers in data mining. The classification effectiveness of KNN relies on the distance metric in the feature space, with the default number of neighbors in the neighbor query set to 5. By observing the impact of different features on classification accuracy, one can directly assess the role of these features in the classification process. Unlike many other classifiers, KNN does not require an explicit training process. It classifies based on the entire training set, allowing for a rapid evaluation of different feature combinations. Moreover, to mitigate bias from a single partition, we adopted k-fold cross-validation as the evaluation method, providing a robust estimate of model performance by averaging performance across multiple random data splits. Here, k was set to 10, dividing the dataset into ten subsets, training on nine subsets while reserving one as the validation set. This approach ensured that each subset had the chance to be the validation set, enabling a comprehensive evaluation of the models’ performance.

### 5.4. Parameter Testing

In the EQIGWO algorithm, the parameter *T* serves as a threshold that controls the transition from exploration to exploitation. The test results for parameter *T* are shown in Table 3. When *T* = 10, the algorithm may not sufficiently explore the search space, leading to premature convergence in a certain region and causing it to become stuck in a local optimum. On the other hand, when *T* = 20, the algorithm emphasizes exploration in the early stages, making it difficult to fine-tune local solutions, which may result in missing the global optimum. Setting *T* = 15 strikes a balance between exploration and exploitation. Compared to *T* = 10, *T* = 15 allows for a longer exploration phase, ensuring that the algorithm searches a broader space and finds more promising solutions. Meanwhile, compared to *T* = 20, setting *T* = 15 enables the algorithm to enter the exploitation phase earlier, focusing on local search sooner and increasing the convergence precision of the algorithm.

### 5.5. The Ablation Experiments

To validate the effectiveness of the proposed enhancements, we conducted validations for each component. We denote the algorithm that adds the linear integration of individual information enhancement exploration strategy on top of GWO as EGWO. EGWO-QI incorporates the population diversity fusion with the local development quadratic interpolation technique into EGWO. DEGWO-QI further integrates chaotic convergence factors into EGWO-QI. Finally, EQIGWO builds upon DEGWO-QI by incorporating the novel transfer function.

Table 4 presents a comparison of the optimal accuracy achieved by the enhancement strategies. From the results, we can observe that all four enhancement strategies were effective. For EGWO, the increased global search capability led to a noticeable improvement in accuracy on most datasets. However, the enhancement in the global search capability inevitably led to a decrease in the local development capability. Therefore, the slight decrease in accuracy on datasets such as Australian, Climate, Fri_c0_500_10, Fri_c1_1000_10, Lymphography, Robot-failures-lp2, Segment, and Vowel was acceptable. Addressing the deficiency in the local development capability of EGWO, EGWO-QI enhanced the alpha wolf’s refinement in development through quadratic interpolation, resulting in a significant increase in classification accuracy. Additionally, the strategy of retaining inferior individuals through quadratic interpolation increased population diversity, leading to substantial performance improvements for EGWO-QI on high-dimensional or large datasets such as Breast Cancer Coimbra, German, HeartEW, Kc1, Lymphography, Robot-failures-lp1, Robot-failures-lp5, SonarEW, and SpectEW. For DEGWO-QI, further enhancement to the global search capability on top of EGWO-QI resulted in improved accuracy on the datasets. EQIGWO increased classification accuracy by incorporating more feature information through a novel transfer function. As a result, EQIGWO achieved the best classification accuracy on all datasets, except for Robot-failures-lp2.

**Figure 4:**
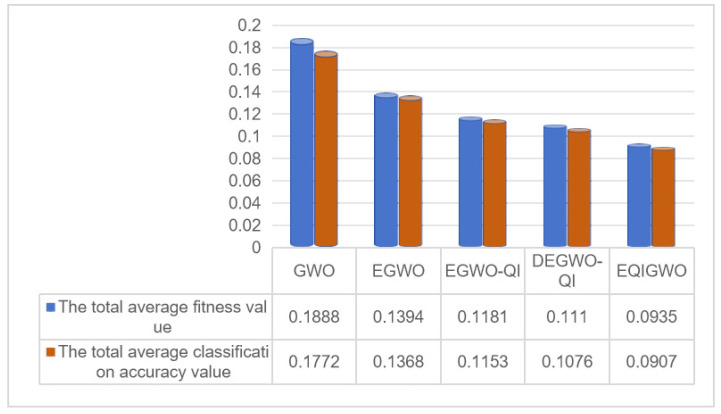
The average classification error rates and fitness values of the algorithms with the successive addition of enhancement strategies across all datasets.

From the comparison results of the optimal fitness achieved by the enhancement strategies in Table 5, it can be observed that the fitness of EGWO was mostly lower than that of the original Gray Wolf Optimization (GWO) algorithm. EGWO-QI addressed the issue of reduced local exploitation capability in EGWO, resulting in a decrease in fitness on 27 datasets, except for Diabetic Retinopathy, Glass, Parkinsons, Page Blocks, and Vehicle. Additionally, compared to EGWO-QI, DEGWO-QI exhibited a decrease in fitness on almost all datasets. Finally, EQIGWO only failed to achieve the best fitness on the Robot-failures-lp2 dataset. Therefore, all four enhancement strategies proposed in this study were effective. Furthermore, in Figure 4, it can be observed that the total average misclassification rates for GWO, EGWO, EGWO-QI, DEGWO-QI, and EQIGWO across all datasets were 0.1886, 0.1531, 0.1311, 0.1246, and 0.1049, respectively, while the total average fitness values across all datasets were 0.2027, 0.1551, 0.1333, 0.1271, and 0.1075, respectively. Both metrics showed a gradual decrease, validating the effectiveness of the four enhancement strategies proposed in this study.

### 5.6. Comparison with Other Intelligence Algorithms

**Figure 5:**
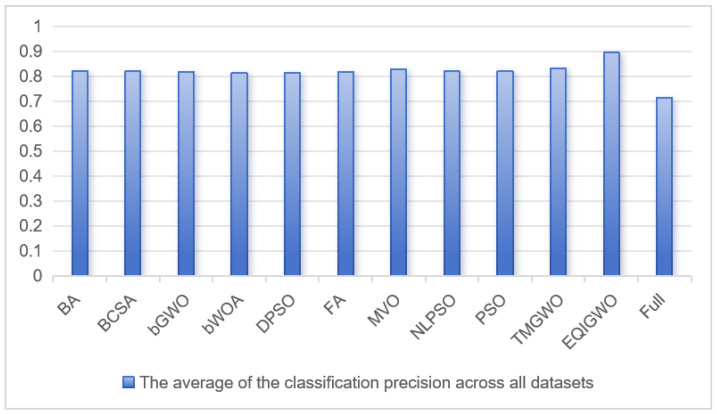
The average classification accuracy of the algorithms across all datasets.

Table 6 provides a comparison of the EQIGWO algorithm with other intelligence algorithms based on accuracy. The “Full” column represents the accuracy without feature selection. The EQIGWO algorithm achieved the highest classification accuracy on 29 out of 32 datasets and slightly lower accuracy than the other algorithms on the remaining 3 datasets. Furthermore, thorough exploration enabled the EQIGWO algorithm to achieve 100% classification accuracy on the Parkinsons and Zoo datasets, which is commendable. Additionally, the EQIGWO algorithm demonstrated significant improvements in accuracy on datasets where other algorithms were prone to local optima, such as Breast Cancer Coimbra, Breast Cancer Tissue, Fri_c0_500_10, German, Glass, HeartEW, Lung Cancer, and WDBC, thanks to its strong global search capability and the strategy of retaining inferior individuals through quadratic interpolation. Moreover, in Figure 5, it can be observed that the EQIGWO algorithm ranked first in overall average classification accuracy with a value of 0.894, while the TMGWO algorithm ranked second with a value of 0.831. Correspondingly, the overall average feature counts for the EQIGWO and TMGWO algorithms were 10.96 and 8.09, respectively. The EQIGWO algorithm achieved higher accuracy with a slightly higher number of features, mitigating the risk of overlooking crucial feature information through the novel transfer function, which effectively expanded the search space of feature information, thereby contributing to better results. The EQIGWO algorithm generally had a slightly higher average number of features compared to the TMGWO algorithm, but it represented a substantial reduction from the original average of 29.78. Therefore, the EQIGWO algorithm significantly reduces feature dimensionality, improves classification accuracy, and successfully addresses the multi-objective problem of feature selection.

**Figure 6:**
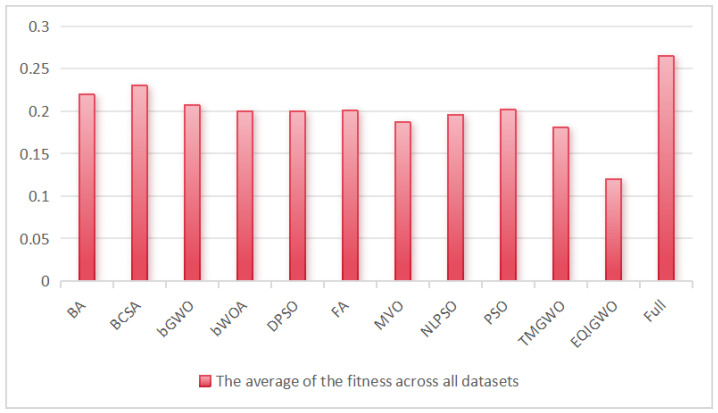
The average fitness values of the algorithms across all datasets.

Table 7 shows a comparison of the proposed algorithm with the other 10 algorithms from the perspective of average fitness, where ‘Full’ represents the accuracy without feature selection. As shown in Figure 6, the proposed algorithm achieved the lowest fitness on 29 datasets, and its overall average fitness was much lower than that of the other algorithms at 0.1204. The experimental results demonstrate significant improvements in the global search capability of the Gray Wolf Optimization algorithm through the linear integration of individual information and the application of the chaotic convergence factor during the exploration phase, thus avoiding premature convergence. Additionally, the quadratic interpolation technique enhanced the algorithm’s utilization, and the strategy of preserving inferior individuals in quadratic interpolation helped the algorithm escape local optima on complex datasets. The statistical results indicate the superiority of our proposed algorithm in terms of classification accuracy and fitness value.

**Figure 7:**
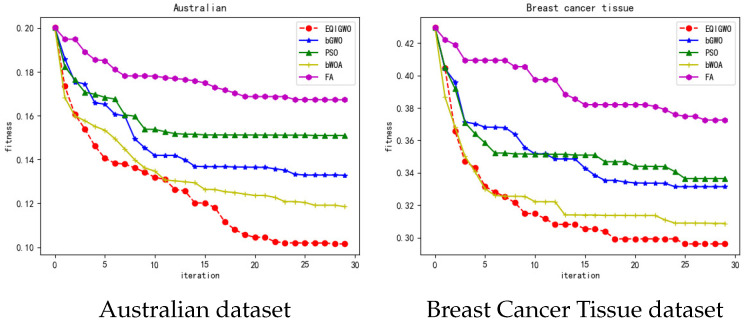
Convergence curves for the Australian and Breast Cancer Tissue datasets.

**Figure 8:**
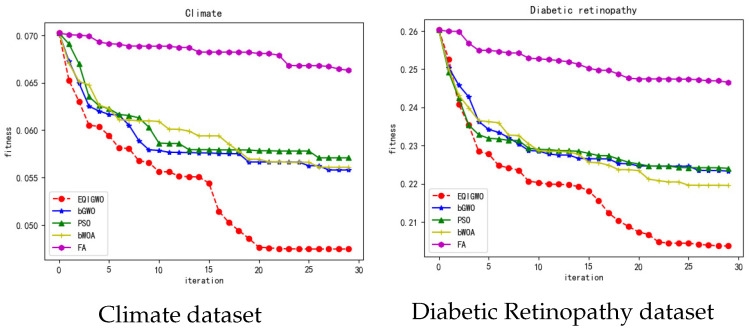
Convergence curves for the Climate and Diabetic Retinopathy datasets.

**Figure 9:**
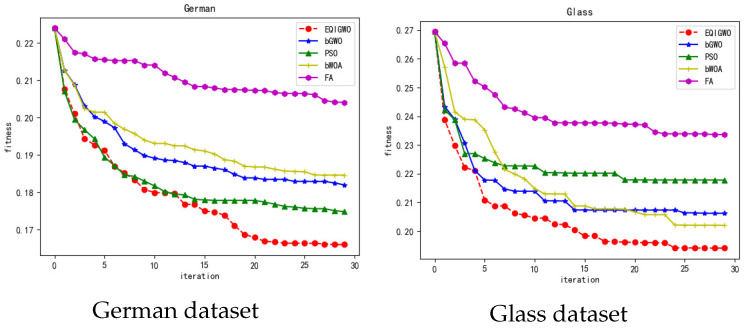
Convergence curves for the German and Glass datasets.

**Figure 10:**
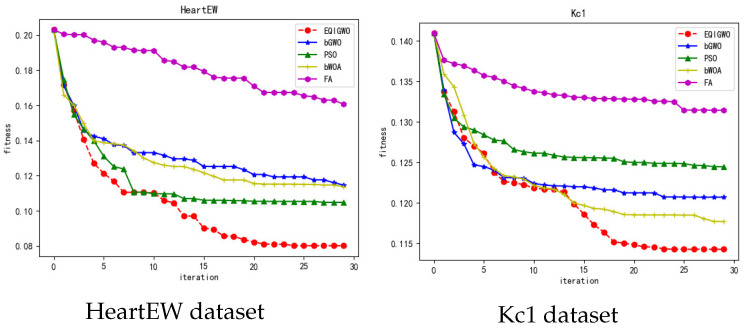
Convergence curves for the HeartEW and Kc1 datasets.

**Figure 11:**
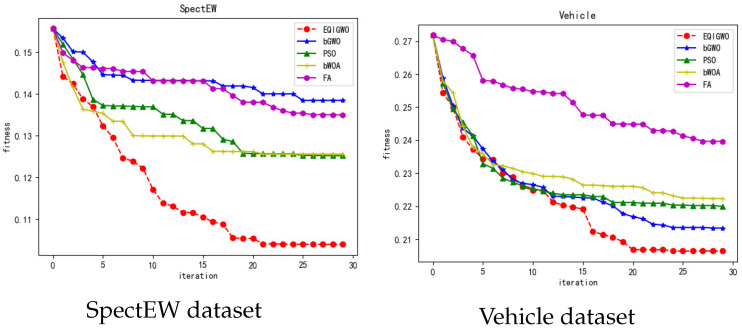
Convergence curves for the SpectEW and Vehicle datasets.

During the training process, we plotted the fitness convergence curves for several algorithms and datasets, as shown in Figure 7, Figure 8, Figure 9, Figure 10 and Figure 11. From these convergence curves, it can be seen that the EQIGWO algorithm’s convergence curve exhibits significant drops after multiple stable phases, indicating that the EQIGWO algorithm demonstrates excellent population diversity and has a strong ability to escape local optima. Furthermore, the EQIGWO algorithm achieved the highest convergence accuracy, which proves its strong local exploitation capability, thus achieving a good balance between diversity and exploitation. Combining the results in Table 6 with the data from the convergence curves, it can be observed that FA (Fruit Fly Algorithm) exhibits the smallest difference in fitness between the training and testing sets, indicating the best generalization ability. This also provides an important direction for the future improvement of the EQIGWO algorithm.

### 5.7. Statistical Test

To test the statistical difference in accuracy between the algorithms, we conducted a *t*-test. Before performing the *t*-test, we used the Shapiro–Wilk test to check the normality of the accuracy data. The results indicated that the data were approximately normally distributed, making it suitable for further analysis using the *t*-test. Table 8 presents the *t*-test results for five randomly selected algorithms and datasets. The *t*-test provides the t-statistic and *p*-value, which help determine whether the performance differences between the two feature selection methods are statistically significant. If the *p*-value is less than 0.05, it indicates a significant difference in accuracy between the two methods, thereby providing a basis for selecting the best feature selection method.

On the Breast Cancer Tissue and WDBC datasets, the t-statistics between the EQIGWO algorithm and the other algorithms were highly significant. All the algorithms had a *p*-value of 0.000, indicating that the differences between the EQIGWO algorithm and these algorithms were statistically highly significant. These results suggest that the EQIGWO algorithm demonstrated a significant advantage in classification accuracy compared to the other algorithms on these datasets. On the Fri_c0_1000_10 and SpectEW datasets, the t-statistics were lower than those on the previous two datasets but still showed significant differences. The t-statistics for the BA algorithm were 2.7917 and 3.0412, respectively; although these values were relatively lower, the *p*-values were 0.008 and 0.004, indicating that the differences were still statistically significant. Compared to the other algorithms, this shows that the EQIGWO algorithm achieved better classification accuracy on these datasets. On the Page Blocks dataset, the t-statistic for the bWOA algorithm was 1.8398, with a *p*-value of 0.073, indicating that the difference between it and the EQIGWO algorithm was not statistically significant, which suggests that these two algorithms performed similarly on this dataset. These results show that the EQIGWO algorithm outperformed the traditional feature selection algorithms in terms of classification accuracy across various datasets. Furthermore, while some algorithms exhibited performance close to that of the EQIGWO algorithm, overall, the EQIGWO algorithm demonstrated strong competitiveness across most datasets.

### 5.8. Complexity Analysis

In this paper, the algorithm’s computational complexity analysis mainly focused on two aspects: space complexity and time complexity. Table 9 presents the average runtimes of the bGWO, EQIGWO, and DEGWO-T algorithms across different datasets to reflect the time complexity of the algorithms, with the optimization time measured in seconds. DEGWO-T is a version of EQIGWO with the “population diversity fused with the local development quadratic interpolation technique” module removed. As indicated in Table 9, the optimization time for DEGWO-T increased only slightly compared to GWO. However, applying quadratic interpolation by dimension means that quadratic polynomial interpolation is computed for each dimension, generating more precise solutions but also increasing the computational complexity during the iteration process. Additionally, retaining inferior individuals increases the number of individuals that must be processed in later iterations. The combination of these two factors led to an increase in runtime. Consequently, EQIGWO required more optimization time than DEGWO-T. However, as shown in the ablation study results in Table 4 and Table 5, adding the “population diversity fused with the local development quadratic interpolation technique” module can significantly improve the convergence accuracy of the algorithm. This module also helps maintain population diversity, preventing the algorithm from prematurely converging to local optima. Although the “population diversity fused with the local development quadratic interpolation technique” module considerably increases runtime, from the perspective of global search capability and local optimization accuracy, this improvement is effective for solving complex optimization problems. Through theoretical analysis and experimental validation, it can be demonstrated that this module enhances population diversity, improves solution accuracy, and helps avoid local optima, thereby enhancing the overall performance of the algorithm.

Space complexity measures the memory required by the algorithm during execution. In the Gray Wolf Optimization algorithm, the initial population size is *N*, and the dimensionality of the search space is *n*. Throughout the optimization process, the population size *N* and dimension *n* remain fixed. Therefore, the space complexity is determined by the need to store the position information of each individual, which can be expressed as O(N×n). In the EQIGWO algorithm, the storage of each individual’s historical best position is added. Each individual needs to store not only its current position but also an additional vector for the historical best position. Hence, each individual requires two position vectors, and the space complexity doubles to O(N×n)+O(N×n). Additionally, the operation of probabilistically retaining weaker individuals increases the population by *M* individuals. Therefore, the overall space complexity of the algorithm becomes O(2×N×n)+O(2×M×n).

## 6. Conclusions

The paper presents a novel Gray Wolf Optimization algorithm called EQIGWO, which addresses several drawbacks of the original GWO algorithm, such as insufficient exploration and overreliance on the alpha wolf, leading to premature convergence, low population diversity, and suboptimal convergence accuracy, including the feature selection problem. The EQIGWO algorithm incorporates four improvements. First, it enhances the algorithm’s global exploration capability by linearly integrating individual information to prevent premature convergence. Second, to increase convergence accuracy while addressing the decrease in local development capability due to the integration of individual information, the EQIGWO algorithm aggressively selects the best current solution at each iteration through quadratic interpolation. When the result of quadratic interpolation is superior, it replaces the alpha wolf. Due to the specificity of the points selected for quadratic interpolation, inferior solutions may be generated. In such cases, the fitness of inferior solutions is substituted into a predefined function. If the result of the function is greater than a random value, the generated inferior solution is inserted into the population. This allows for the exploration of less favorable regions, thereby increasing population diversity and potentially leading to the discovery of better global solutions. Furthermore, the EQIGWO algorithm introduces chaotic perturbation to the convergence factor during the exploration phase to further enhance the exploration capability. Finally, the new transfer function ensures that critical information is retained during the early stages. In the middle stages, it prevents the addition of irrelevant feature information, while in the later stages, it increases the probability of selecting non-critical feature information to avoid the loss of essential data.

The algorithm was tested on 32 datasets, and the EQIGWO algorithm’s classification accuracy and fitness were superior to those of the original GWO algorithm on 31 datasets. Additionally, compared with 10 other advanced algorithms, the EQIGWO algorithm achieved the lowest fitness and highest classification accuracy on 29 datasets, demonstrating the outstanding effectiveness of the improvement strategies proposed in this paper. Furthermore, from *t*-tests conducted on multiple datasets, the results show that the EQIGWO algorithm demonstrated significant advantages over traditional algorithms across various types of datasets. This further proves the effectiveness and adaptability of the EQIGWO algorithm in addressing feature selection problems. Although the GWO algorithm achieved the best classification accuracy, it did not minimize the number of selected features. This indicates that, although it improved model performance, the GWO algorithm selected more features, leading to increased computational costs. To address this issue, we plan to propose a new transfer function aimed at more precisely evaluating the importance of feature information, thereby more effectively addressing the problem of feature redundancy. Moreover, experimental results showed that the EQIGWO algorithm demonstrated outstanding performance in feature selection tasks. As data dynamically evolves (e.g., time-series data or real-time streaming data), the importance of features may fluctuate over time. Future research could combine dynamic optimization algorithms with reinforcement learning, enabling models to dynamically adjust the selected features according to changes in the data, thereby improving the adaptability and performance of the model. 

## Figures and Tables

**Table 1:** Description of datasets.

Dataset	Instances	Samples	Classes
Australian	14	690	2
Breast Cancer Coimbra	9	116	2
Breast Cancer Tissue	9	106	6
Climate	20	540	2
Diabetic Retinopathy	19	1151	2
Fri_c0_500_10	10	500	2
Fri_c0_1000_10	10	1000	2
Fri_c1_1000_10	10	1000	2
German	24	1000	2
Glass	9	2145	7
HeartEW	13	270	5
IonosphereEW	34	351	2
Kc1	21	2109	2
Kc2	21	522	2
Lung Cancer	21	226	2
Lymphography	18	148	4
Page Blocks	10	5473	2
Parkinsons	22	195	2
Pc1	21	1109	2
Robot-failures-lp1	90	88	4
Robot-failures-lp2	90	47	5
Robot-failures-lp3	90	47	4
Robot-failures-lp4	90	117	3
Robot-failures-lp5	90	164	5
Segment	19	2310	7
SonarEW	60	208	2
SpectEW	22	267	2
Vehicle	18	846	4
Vowel	10	990	2
WineEW	13	178	3
WDBC	30	569	2
Zoo	16	101	7

**Table 2:** Parameter settings.

Algorithm	Parameter Settings
BA	$F_{\min}=0,\;F_{\max}=2,\;A=0.25,\;r=0.5,\;\varepsilon =\left[-1,\;1\right],\;\alpha =0.9,\;\gamma =0.9$
BCSA	AP=0.1, fl=2.0
bGWO	α decreases linearly from 2 to 0
bWOA	α decreases linearly from 2 to 0
DPSO	$C_{1}=2,\;C_{2}=1.5,\;C_{3}=0.5,\;\omega_{\max}=0.995,\;\omega_{\min}=0.5$
FA	p=0.8
MVO	min=0.2, max=1,p=6
NLPSO	μ=0.2ϕ=0.05λ=0.5σ=10
PSO	$\omega_{1}=2,\;\omega_{2}=2$
TMGWO	$M_{p}=0.5$

**Table 3:** The results of the parameter testing.

Data	*T* = 10	*T* = 20	*T* = 15	Data	*T* = 10	*T* = 20	*T* = 15
Australian	0.852	0.860	**0.871**	Parkinsons	**1.000**	**1.000**	**1.000**
Breast Cancer Coimbra	0.921	0.893	**0.932**	Page Blocks	0.961	**0.972**	0.968
Breast Cancer Tissue	0.632	0.652	**0.670**	Pc1	0.940	0.942	**0.947**
Climate	0.940	**0.946**	**0.946**	Robot-failures-lp1	0.914	**0.943**	0.930
Diabetic Retinopathy	0.742	0.751	**0.759**	Robot-failures-lp2	0.665	0.670	**0.735**
Fri_c0_500_10	0.871	0.890	**0.900**	Robot-failures-lp3	**0.745**	0.720	0.735
Fri_c0_1000_10	0.871	0.872	**0.885**	Robot-failures-lp4	0.922	0.934	**0.946**
Fri_c1_1000_10	0.909	0.917	**0.921**	Robot-failures-lp5	0.760	0.810	**0.816**
German	0.780	0.789	**0.807**	Segment	0.964	0.967	**0.973**
Glass	0.761	0.793	**0.821**	SonarEW	0.924	0.939	**0.951**
HeartEW	0.880	0.847	**0.903**	SpectEW	0.869	0.885	**0.894**
IonosphereEW	0.915	**0.942**	0.940	Vehicle	0.753	0.753	**0.756**
Kc1	0.870	0.870	**0.876**	Vowel	0.988	0.995	**0.996**

**Table 4:** Comparison of accuracy achieved by different enhancement strategies.

Name	GWO	EGWO	EGWO-QI	DEGWO-QI	EQIGWO
Australian	0.836	0.810	0.837	0.837	**0.871**
Breast Cancer Coimbra	0.736	0.795	0.835	0.846	**0.932**
Breast Cancer Tissue	0.330	0.565	0.584	0.598	**0.670**
Climate	0.920	0.914	0.938	0.938	**0.946**
Diabetic Retinopathy	0.700	0.728	0.728	0.728	**0.759**
Fri_c0_500_10	0.866	0.860	0.869	0.876	**0.900**
Fri_c0_1000_10	0.861	0.870	0.873	0.874	**0.885**
Fri_c1_1000_10	0.897	0.884	0.896	0.916	**0.921**
German	0.741	0.754	0.805	0.788	**0.807**
Glass	0.738	0.738	0.722	0.770	**0.821**
HeartEW	0.814	0.825	0.896	0.900	**0.903**
IonosphereEW	0.879	0.919	0.924	0.928	**0.940**
Kc1	0.693	0.829	0.862	0.868	**0.876**
Kc2	0.771	0.848	0.874	0.880	**0.887**
Lung Cancer	0.886	0.912	0.916	0.937	**0.945**
Lymphography	0.864	0.830	0.905	0.918	**0.952**
Parkinsons	0.857	0.984	0.984	0.984	**1.000**
Page Blocks	0.956	0.965	0.965	0.965	**0.968**
Pc1	0.914	0.938	0.944	0.944	**0.947**
Robot-failures-lp1	0.875	0.898	0.920	0.918	**0.930**
Robot-failures-lp2	**0.750**	0.689	0.784	0.695	0.735
Robot-failures-lp3	0.700	0.700	0.720	0.720	**0.735**
Robot-failures-lp4	0.881	0.893	0.896	0.913	**0.946**
Robot-failures-lp5	0.631	0.741	0.763	0.779	**0.816**
Segment	0.968	0.964	0.966	0.970	**0.973**
SonarEW	0.605	0.874	0.927	0.937	**0.951**
SpectEW	0.742	0.815	0.875	0.875	**0.894**
Vehicle	0.725	0.726	0.739	0.741	**0.756**
Vowel	0.987	0.974	0.972	0.989	**0.996**
WineEW	0.935	0.950	0.950	0.964	**0.994**
WDBC	0.948	0.950	0.957	**0.961**	**0.961**
Zoo	0.960	0.960	0.980	0.980	**1.000**

**Table 5:** Comparison of fitness values achieved by different enhancement strategies.

Name	GWO	EGWO	EGWO-QI	DEGWO-QI	EQIGWO
Australian	0.1664	0.1935	0.1645	0.1645	**0.1307**
Breast Cancer Coimbra	0.2654	0.2072	0.1675	0.1559	**0.0709**
Breast Cancer Tissue	0.6677	0.4347	0.4159	0.3516	**0.3304**
Climate	0.0828	0.0876	0.0639	0.0652	**0.0567**
Diabetic Retinopathy	0.3017	0.2735	0.2737	0.2732	**0.2427**
Fri_c0_500_10	0.1376	0.1432	0.1347	0.1288	**0.1043**
Fri_c0_1000_10	0.1436	0.1333	0.1316	0.1302	**0.1191**
Fri_c1_1000_10	0.1059	0.1185	0.1072	0.0878	**0.0789**
German	0.2643	0.2479	0.1974	0.2144	**0.1962**
Glass	0.2637	0.2632	0.2798	0.2041	**0.1812**
HeartEW	0.1894	0.1765	0.1058	0.1028	**0.0997**
IonosphereEW	0.1226	0.0824	0.0782	0.0740	**0.0623**
Kc1	0.3093	0.1730	0.1405	0.1346	**0.1261**
Kc2	0.2337	0.1532	0.1274	0.1225	**0.1101**
Lung Cancer	0.1177	0.0895	0.0858	0.0650	**0.0579**
Lymphography	0.1421	0.1713	0.0970	0.0846	**0.0504**
Parkinsons	0.1452	0.0203	0.0214	0.0218	**0.0052**
Page Blocks	0.4066	0.0363	0.0366	0.0363	**0.0315**
Pc1	0.0887	0.0635	0.0581	0.0575	**0.0550**
Robot-failures-lp1	0.1298	0.1038	0.0819	0.0844	**0.0728**
Robot-failures-lp2	**0.2513**	0.3103	0.2165	0.3053	0.2658
Robot-failures-lp3	0.3006	0.2995	0.2803	0.2810	**0.2659**
Robot-failures-lp4	0.1211	0.1089	0.1060	0.0900	**0.0571**
Robot-failures-lp5	0.3703	0.2595	0.2382	0.2229	**0.1864**
Segment	0.0301	0.0397	0.0378	0.0340	**0.0313**
SonarEW	0.3962	0.1281	0.0759	0.0661	**0.0433**
SpectEW	0.2623	0.1861	0.1267	0.1285	**0.1090**
Vehicle	0.2794	0.2764	0.2627	0.2611	**0.2373**
Vowel	0.0189	0.0317	0.0339	0.0168	**0.0102**
WineEW	0.0717	0.0565	0.0525	0.0387	**0.0092**
WDBC	0.0562	0.0525	0.0454	0.0418	**0.0418**
Zoo	0.0452	0.0416	0.0216	0.0220	**0.0019**

**Table 6:** Comparison of the accuracy of the EQIGWO algorithm and other algorithms.

Data	BA	BCSA	bGWO	bWOA	DPSO	FA	MVO	NLPSO	PSO	TMGWO	EQIGWO	Full
Australian	0.831	0.830	0.836	0.839	0.831	0.837	0.831	0.833	0.830	0.846	**0.871**	0.685
Breast Cancer Coimbra	0.736	0.736	0.736	0.727	0.736	0.736	0.736	0.736	0.736	0.736	**0.932**	0.381
Breast Cancer Tissue	0.330	0.330	0.330	0.320	0.330	0.330	0.330	0.320	0.330	0.330	**0.670**	0.200
Climate	0.918	0.941	0.924	0.922	0.918	0.927	0.927	0.920	0.941	0.931	**0.946**	0.888
Diabetic Retinopathy	0.924	0.679	0.925	0.704	0.693	0.708	0.711	0.694	0.697	0.712	**0.759**	0.344
Fri_c0_500_10	0.850	0.866	0.836	0.866	0.854	0.866	0.866	0.866	0.866	0.866	**0.900**	0.799
Fri_c0_1000_10	0.875	0.875	0.861	0.875	0.875	0.875	0.875	0.875	0.875	0.875	**0.885**	0.787
Fri_c1_1000_10	0.901	0.901	0.901	0.897	0.901	0.901	0.901	0.897	0.901	0.901	**0.921**	0.775
German	0.742	0.746	0.744	0.740	0.743	0.746	0.754	0.742	0.746	0.756	**0.807**	0.685
Glass	0.734	0.738	0.738	0.738	0.738	0.738	0.738	0.738	0.738	0.738	**0.821**	0.695
HeartEW	0.825	0.825	0.840	0.814	0.840	0.840	0.829	0.822	0.825	0.840	**0.903**	0.662
IonosphereEW	0.876	0.908	0.882	0.894	0.900	0.905	0.905	0.914	0.908	0.931	**0.940**	0.828
Kc1	0.700	0.696	0.702	0.697	0.693	0.692	0.703	0.694	0.696	0.704	**0.876**	0.693
Kc2	0.784	0.807	0.773	0.775	0.803	0.788	0.792	0.796	0.807	0.796	**0.887**	0.732
Lung Cancer	0.877	0.872	0.886	0.877	0.877	0.877	0.900	0.877	0.872	0.900	**0.945**	0.831
Lymphography	0.877	0.878	0.864	0.871	0.871	0.857	0.885	0.878	0.878	0.900	**0.952**	0.771
Parkinsons	0.857	0.868	0.857	0.857	0.868	0.857	0.863	0.868	0.868	0.868	**1.000**	0.742
Page Blocks	0.916	0.955	0.963	0.964	0.961	0.961	0.961	0.964	0.957	0.963	**0.968**	0.953
Pc1	0.916	0.916	0.914	0.917	0.915	0.915	0.917	0.914	0.916	0.917	**0.947**	0.901
Robot-failures-lp1	0.892	0.887	0.875	0.875	0.862	0.875	0.912	0.887	0.887	0.912	**0.930**	0.762
Robot-failures-lp2	0.750	0.775	0.725	0.725	0.750	0.750	0.775	0.750	0.775	0.775	0.735	0.675
Robot-failures-lp3	0.725	0.750	0.725	0.725	0.750	0.750	0.750	0.750	0.750	0.775	0.735	0.575
Robot-failures-lp4	0.875	0.881	0.872	0.881	0.872	0.890	0.927	0.900	0.881	0.936	**0.946**	0.809
Robot-failures-lp5	0.637	0.650	0.625	0.631	0.631	0.643	0.687	0.637	0.625	0.675	**0.816**	0.593
Segment	0.971	0.967	0.970	0.967	0.968	0.970	0.974	0.965	0.967	0.974	0.973	0.956
SonarEW	0.665	0.679	0.620	0.660	0.675	0.660	0.730	0.680	0.679	0.740	**0.951**	0.505
SpectEW	0.742	0.734	0.750	0.723	0.740	0.734	0.757	0.734	0.734	0.761	**0.894**	0.680
Vehicle	0.726	0.732	0.729	0.730	0.725	0.727	0.734	0.726	0.732	0.738	**0.756**	0.657
Vowel	0.988	0.953	0.888	0.967	0.898	0.988	0.988	0.988	0.975	0.988	**0.996**	0.735
WineEW	0.941	0.935	0.941	0.941	0.941	0.941	0.946	0.935	0.935	0.947	**0.994**	0.664
WDBC	0.947	0.946	0.948	0.948	0.948	0.948	0.948	0.948	0.946	0.948	**0.961**	0.925
Zoo	0.960	0.960	0.960	0.960	0.960	0.960	0.960	0.960	0.960	0.940	**1.000**	0.940
Total mean	0.821	0.819	0.816	0.813	0.814	0.818	0.828	0.819	0.819	0.831	**0.894**	0.713

**Table 7:** Comparison of the fitness values of the EQIGWO algorithm and other algorithms.

Data	BA	BCSA	bGWO	bWOA	DPSO	FA	MVO	NLPSO	PSO	TMGWO	EQIGWO	Full
Australian	0.1781	0.2220	0.2007	0.1882	0.1813	0.1819	0.1759	0.1813	0.1900	0.1686	**0.1493**	0.3213
Breast Cancer Coimbra	0.2812	0.3253	0.2713	0.2811	0.2769	0.2808	0.2707	0.2783	0.2799	0.2672	**0.1172**	0.6220
Breast Cancer Tissue	0.6955	0.7313	0.6866	0.7007	0.6909	0.6844	0.6788	0.6893	0.7014	0.6817	**0.3622**	0.8020
Climate	0.0849	0.0774	0.0870	0.0845	0.0854	0.0827	0.0844	0.0686	0.0861	0.0804	**0.0569**	0.1199
Diabetic Retinopathy	0.3175	0.3276	0.3160	0.3161	0.3175	0.3114	0.3013	0.3128	0.3159	0.3011	**0.2547**	0.3440
Fri_c0_500_10	0.1531	0.1728	0.1703	0.1579	0.1584	0.1405	0.1513	0.1548	0.1546	0.1376	**0.1203**	0.2080
Fri_c0_1000_10	0.1628	0.1828	0.1574	0.1518	0.1476	0.1585	0.1372	0.1510	0.1560	**0.1287**	0.1311	0.2208
Fri_c1_1000_10	0.1148	0.1594	0.1280	0.1238	0.1190	0.1199	0.1037	0.1134	0.1224	0.1033	**0.0903**	0.2327
German	0.2733	0.2835	0.2711	0.2713	0.2699	0.2688	0.2642	0.2681	0.2701	0.2592	**0.2114**	0.3208
Glass	0.2691	0.2741	0.2682	0.2683	0.2672	0.2678	0.2652	0.2680	0.2687	0.2647	**0.2071**	0.3117
HeartEW	0.1988	0.2318	0.2051	0.2089	0.1874	0.2047	0.1955	0.2009	0.2155	0.1760	**0.1209**	0.3436
IonosphereEW	0.1257	0.1202	0.1339	0.1190	0.1128	0.1146	0.1118	0.1029	0.1175	0.0906	**0.0678**	0.1797
Kc1	0.3088	0.3190	0.3103	0.3101	0.3098	0.3120	0.3040	0.3094	0.3115	0.3029	**0.1379**	0.3329
Kc2	0.7846	0.2333	0.2385	0.2339	0.2259	0.2296	0.2187	0.2168	0.2253	0.2235	**0.1195**	0.2746
Lung Cancer	0.1360	0.4715	0.1352	0.1369	0.1334	0.1342	0.1211	0.1335	0.1370	0.1172	**0.0619**	0.1765
Lymphography	0.1587	0.1796	0.1608	0.1531	0.1477	0.1577	0.1441	0.1502	0.1615	0.1400	**0.0812**	0.2365
Parkinsons	0.1815	0.1945	0.1642	0.1555	0.1536	0.1651	0.1479	0.1497	0.1615	0.1404	**0.0065**	0.0563
Page Blocks	0.0458	0.0509	0.0475	0.0485	0.0482	0.0472	0.0455	0.0485	0.0469	0.0455	**0.0337**	0.2653
Pc1	0.0896	0.0925	0.0918	0.0904	0.0902	0.0899	0.0878	0.0890	0.0908	0.0869	**0.0552**	0.1071
Robot-failures-lp1	0.1464	0.2014	0.1532	0.1487	0.1439	0.1412	0.1138	0.1448	0.1403	0.0909	**0.0826**	0.2451
Robot-failures-lp2	0.2739	0.2903	0.2812	0.2835	0.2741	0.2759	0.2489	0.2671	0.2657	0.2415	**0.2867**	0.3317
Robot-failures-lp3	0.3026	0.3255	0.3043	0.3061	0.2821	0.3058	0.2716	0.2902	0.3056	**0.2493**	0.2789	0.4307
Robot-failures-lp4	0.1464	0.1563	0.1442	0.1400	0.1372	0.1405	0.0912	0.1296	0.1303	0.0826	**0.0616**	0.1989
Robot-failures-lp5	0.3860	0.4043	0.3893	0.3866	0.3888	0.3849	0.3460	0.3858	0.3874	0.3430	**0.1907**	0.4121
Segment	0.0381	0.0464	0.0405	0.0419	0.0401	0.0413	0.0311	0.0396	0.0415	**0.0284**	0.0351	0.0532
SonarEW	0.3957	0.3914	0.4161	0.3876	0.3584	0.4055	0.3347	0.3616	0.3795	0.3337	**0.0537**	0.5000
SpectEW	0.2858	0.3053	0.2835	0.2880	0.2816	0.2876	0.2727	0.2826	0.2833	0.2644	**0.1316**	0.3260
Vehicle	0.2885	0.3074	0.2866	0.2833	0.2884	0.2868	0.2808	0.2830	0.2855	0.2731	**0.2497**	0.3494
Vowel	0.0109	0.0437	0.1012	0.0313	**0.1001**	0.0109	0.0109	0.0109	0.0224	0.0109	0.0164	0.2720
WineEW	0.0824	0.1211	0.0771	0.0800	0.0706	0.0742	0.0706	0.0788	0.0798	0.0638	**0.0299**	0.0843
WDBC	0.0602	0.0700	0.0607	0.0598	0.0587	0.0604	0.0559	0.0596	0.0605	0.0535	**0.0447**	0.3419
Zoo	0.0541	0.0570	0.0452	0.0517	0.0505	0.0518	0.0488	0.0515	0.0529	0.0485	**0.0062**	0.0842
Total mean	0.2197	0.2303	0.2070	0.2002	0.1999	0.2005	0.1870	0.1959	0.2014	0.1812	**0.1204**	0.2651

**Table 8:** The results of the statistical test.

Dataset	Algorithm	t_statistic	*p*-Value
Breast Cancer Tissue	BA	12.488	0.000
	bGWO	18.678	0.000
	bWOA	11.605	0.000
	PSO	14.866	0.000
	FA	20.645	0.000
WDBC	BA	7.4385	0.000
	bGWO	8.7601	0.000
	bWOA	6.5093	0.000
	PSO	6.2493	0.000
	FA	9.7756	0.000
Fri_c0_1000_10	BA	2.7917	0.008
	bGWO	4.1033	0.002
	bWOA	4.9154	0.000
	PSO	3.6942	0.000
	FA	4.4329	0.000
SpectEW	BA	3.0412	0.004
	bGWO	4.1144	0.000
	bWOA	10.359	0.000
	PSO	12.453	0.000
	FA	9.5893	0.000
Page Blocks	BA	47.674	0.000
	bGWO	3.6322	0.009
	bWOA	1.8398	0.073
	PSO	8.5761	0.000
	FA	10.774	0.000

**Table 9:** Time complexity results.

Data	bGWO	EQIGWO	DEGWO-T	Data	bGWO	EQIGWO	DEGWO-T
Australian	4.88	13.2	4.94	Parkinsons	2.43	7.89	2.61
Breast Cancer Coimbra	4.71	13.7	4.73	Page Blocks	29.9	86.7	30.7
Breast Cancer Tissue	1.95	5.47	1.99	Pc1	7.48	24.1	7.53
Climate	4.28	16.0	4.37	Robot-failures-lp1	4.49	13.6	5.34
Diabetic Retinopathy	7.98	25.1	9.1	Robot-failures-lp2	4.21	13.2	4.91
Fri_c0_500_10	4.04	11.2	4.26	Robot-failures-lp3	4.18	14.4	4.83
Fri_c0_1000_10	6.79	20.4	7.00	Robot-failures-lp4	4.65	14.4	5.33
Fri_c1_1000_10	6.46	18.9	6.61	Robot-failures-lp5	4.98	14.7	5.69
German	7.67	24.3	7.93	Segment	20.1	57.8	21.9
Glass	2.32	6.95	2.36	SonarEW	4.59	14.1	4.71
HeartEW	2.67	7.97	2.72	SpectEW	2.75	8.67	2.87
IonosphereEW	4.52	13.6	4.63	Vehicle	5.60	16.9	6.10
Kc1	13.9	46.3	14.2	Vowel	6.07	17.5	6.16
Kc2	4.32	13.9	4.44	WineEW	2.18	6.06	2.26
Lung Cancer	2.48	7.79	2.57	WDBC	5.48	16.5	5.67
Lymphography	2.07	5.82	2.16	Zoo	1.85	5.66	2.02

## Data Availability

The data that support the findings of this study are available on request from the corresponding author.
